# Phenolic compounds, essential oil composition, and antioxidant activity of Angelica purpurascens (Avé-Lall.) Gill

**DOI:** 10.3906/kim-2101-28

**Published:** 2021-06-30

**Authors:** Semra ALKAN TÜRKUÇAR, Ayça AKTAŞ KARAÇELİK, Mustafa KARAKÖSE

**Affiliations:** 1 Department of Nutrition and Dietetics, Faculty of Health Sciences, Alanya Alaaddin Keykubat University, Antalya Turkey; 2 Department of Food Processing, Espiye Vocational School, Giresun University, Giresun Turkey; 3 Department of Medicinal and Aromatic Plants, Espiye Vocational School, Giresun University, Giresun Turkey

**Keywords:** *Angelica purpurascens*, phenolic compounds, LC−MS/MS, GC−MS, antioxidant activity, total phenolic content, essential oil

## Abstract

In this study, methanol extracts (MEs) and essential oil (EO) of *Angelica purpurascens* (Avé-Lall.) Gill obtained from different parts (root, stem, leaf, and seed) were evaluated in terms of antioxidant activity, total phenolics, compositions of phenolic compound, and essential oil with the methods of 2,2-azino-bis(3ethylbenzo-thiazoline-6-sulfonic acid (ABTS•^+^), 2,2-diphenyl-1-picrylhydrazil (DPPH•) radical scavenging activities, and ferric reducing/antioxidant power (FRAP), the Folin–Ciocalteu, liquid chromatography−tandem mass spectrometry (LC−MS/MS), and gas chromatography-mass spectrometry (GC−MS), respectively. The root extract of *A. purpurascens* exhibited the highest ABTS•^+^, DPPH•, and FRAP activities (IC_50_: 0.05 ± 0.0001 mg/mL, IC_50_: 0.06 ± 0.002 mg/mL, 821.04 ± 15.96 µM TEAC (Trolox equivalent antioxidant capacity), respectively). Moreover, EO of *A. purpurascens* root displayed DPPH• scavenging activity (IC_50_: 2.95 ± 0.084 mg/mL). The root extract had the highest total phenolic content (438.75 ± 16.39 GAE (gallic acid equivalent), µg/mL)). Twenty compounds were identified by LC−MS/MS. The most abundant phenolics were ferulic acid (244.39 ± 15.64 μg/g extract), benzoic acid (138.18 ± 8.84 μg/g extract), oleuropein (78.04 ± 4.99 μg/g extract), and rutin (31.21 ± 2.00 μg/g extract) in seed, stem, root, and leaf extracts, respectively. According to the GC−MS analysis, the major components were determined as α-bisabolol (22.93%), cubebol (14.39%), α-pinene (11.63%), and α-limonene (9.41%) among 29 compounds. Consequently, the MEs and EO of *A. purpurascens* can be used as a natural antioxidant source.

## 1. Introduction

Antioxidants are molecules that can decrease or eliminate the effect of reactive oxygen species (ROS) created as a result of biochemical processes [1–3]. When ROS increases in the body, the oxidative balance is disturbed and causes “oxidative stress” [4]. Oxidative stress causes severe cell damage resulting in aging and various illnesses such as atherosclerosis, asthma, Parkinson’s, cancer, Alzheimer’s, inflammation, and rheumatoid arthritis [5–7]. Synthetic and natural antioxidants are used to preserve the cell from the negative effects of ROS by slowing down or preventing the oxidation process [8,9]. Since high doses of synthetic antioxidants have toxic and carcinogenic effects in animals, natural antioxidants obtained from plants have recently become more preferred over synthetic antioxidants due to their safety and lack of unwanted side effects [1,10].

The genus *Angelica* L *.* belonging to the family Apiaceae has been grown in Asia, Europe, North America, and Africa [11,12]. The genus *Angelica* is extremely rich in secondary metabolites such as flavonoid [13], coumarin [14–16], acetylenic compound [15], sesquiterpene lactones [17], and essential oils [18–20]. The majority of these species are used to strengthen the immune, circulatory, respiratory, and nervous systems and to treat bronchial ailments, colds, urinary sepsis, and indigestion, tumors, as well as in the food industry [21–25] due to their biological activities, including antibacterial, antifungal, insecticidal, and antioxidant activities [26–29]. The most interesting feature of the family is the high chemical diversity of many members, including different aromatic chemicals in different organs such as fruits, flowers, leaves, roots, and stems. The chemical diversity in the underground and aboveground parts of the plant is so different that the essential oil components of the plant can significantly vary [23]. In previous studies, sterols such as xanthogonine, xanthogalol acetate, xanthogalol, xanthalin, ostruthol, isooxyposedan, β-sitosterol, coumarin, agacillin, and acyl- and pyranocoumarins were found in different plant tissues of *Angelica* species [30–32]. 

*A. purpurascens* (Avé-Lall.) Gilli known as ‘melekotu’ in Turkey is the synonym of *Xanthogalum purpurascens* Avé-Lall. [24]. *A. purpurascens* is widely grown in Turkey, especially in the Northeastern Black Sea region [12].

The literature review reveals that although there are many studies on the composition of the essential oils and biological activities of *Angelica* species, the data about *A. purpurascens* essential oil composition and antioxidant activities are limited. However, to the best of our knowledge, there are no reports in the literature regarding its phenolic constituents. 

The current study aims (i) to study the antioxidant capacity of *A. purpurascens* methanol extracts prepared from root, stem, leaf, and seed and the essential oil obtained from the root of *A. purpurascens* using three common methods DPPH•, ABTS•^+^, and FRAP, (ii) to explore the total phenolic contents (TPC) of methanol extracts, (iii) to evaluate the phenolic composition in different parts (root, stem, leaf, and seed) of *A. purpurascens* by LC-MS/MS, and (iv) to determine the chemical composition of essential oil of *A. purpurascens* root by gas chromatography-mass spectroscopy (GC-MS).

## 2. Materials and methods

### 2.1. Chemicals and reagents

2,2’-azino-bis(3-ethylbenzothiazoline-6-sulphonic acid) diammonium salt (ABTS) ≥ 98% (HPLC) (Sigma Aldrich), 2,2-diphenyl-1-picrylhydrazyl (DPPH•) (St. Luis, USA), 2,4,6-tripyridyl-s-triazine (TPTZ) (St. Luis, USA), 6-hydroxy-2,5,7,8-tetramethylchroman-2-carboxylic acid (Trolox) (St. Luis, USA), anhydrous iron (III) chloride (FeCl_3_) (St. Luis, USA), Folin–Ciocalteu reagent (FCR) (St. Luis, USA), anhydrous sodium sulfate (Darmstadt, Germany), sodium hydroxide (Darmstadt, Germany), sodium carbonate (Darmstadt, Germany), acetic acid (Darmstadt, Germany), hydrochloric acid (Darmstadt, Germany), methanol (Darmstadt, Germany), ethanol (Darmstadt, Germany), hexane (Darmstadt, Germany), butylated hydroxytoluene (Darmstadt, Germany), gallic acid (St. Luis, USA), catechin (Sigma Aldrich), quercetin (St. Luis, USA), all HPLC standards (pyrogallol, gallic acid, protocatechuic aldehyde, chlorogenic acid, syringic acid, caffeic acid, 4-hydroxybenzaldehyde, vanillin, syringaldehyde, ferulic acid, hesperidin, luteolin-7-glucoside, rutin, oleuropein, benzoic acid, resveratrol, myricetin, apigenin, naringenin, ellagic acid) were supplied from Sigma Aldrich.

### 2.2. Plant material and sample preparation

*A. purpurascens* was harvested from Alucra, Giresun (Coordinates: 40°31′53″ N; 38°36′03″ E, 1604 m, Alucra district, Çakrak village, Riparian side with Alnus glutinosa gallery forest, 15.09.2019) and identified by a botanist, Dr. Mustafa Karaköse, at Giresun University. The plant samples were enumerated (Collector number: M. Karaköse 1407) in Giresun University Espiye Vocational School Herbarium and recorded.

The root, leaf, seed, and stem parts of the plant were dried at 40 °C for 2 days in an oven and powdered with a laboratory blender. Each part of the plant was weighed 5 or 10 g. All weighed samples were extracted with 50 or 100 mL methanol under reflux at 200 rpm and 40 °C for about 2 h [33], and the extracts were filtered with filter paper and then were centrifuged at 6000 rpm for 15 min. Supernatant fractions were filtered by using 0.45-µm syringe filters (International Ltd., Kent, England) to produce clear extract solutions. A rotary evaporator was used to evaporate the solvents, and final concentrations were adjusted to 10 mg/mL. Extraction yields of methanol extracts of different parts of the plant were 0.4888 g (9.776%) for root, 0.4781 g (9.562%) for leaf, 0.3145 g (6.29%) for seed, and 0.1951 g (3.902%) for stem. The prepared extracts were stored at –18 °C until used for analysis. The EO was hydrodistilled from the ground roots (80 g) of the plant for 5 h by using a Clevenger type apparatus and dehydrated with anhydrous sodium sulfate. The EO obtained with a yield of 0.8125% was stored at +4 °C for further studies.

### 2.3. In vitro antioxidant activity

The antioxidant activities of *A. purpurascens* MEs for each part (root, leaf, seed, and stem) and EO were tested by using three common methods ABTS•^+^ and DPPH• radical scavenging assays and ferric reducing/antioxidant power (FRAP) assay.

#### 2.3.1. Ferric reducing/antioxidant power (FRAP) assay

The FRAP reagent solution was prepared by mixing 10 mM TPTZ, 300 mM, acetate buffer (pH 3.6), and 20 mM FeCl_3_ (1:10:1) [34]. The bottle was then wrapped in aluminum foil and stored at room temperature until analysis was performed. The FRAP reagent was prepared fresh. All extracts were diluted to a concentration of 5 mg/mL. After that, 1.5 mL FRAP reagent was pipetted into a 50-μL sample and mixed. The absorbances were read at 595 nm (UV−Vis spectrophotometer, ATI/Unicam UV2) after incubation of samples for 20 min at room temperature. The calibration graph was obtained by using Trolox (62.5−1000 μM), and antioxidant activity was given as Trolox Equivalent Antioxidant Capacity (μM TEAC).

#### 2.3.2. DPPH• radical scavenging assay 

A 100 µM solution of DPPH• was prepared in methanol, and the solution bottle was wrapped in aluminum foil. Next, the solution was mixed in the magnetic stirrer for at least 1 h. The DPPH• radical scavenging method was used as described by Cuendet et al. (1997) with a few modifications [35]. Five different concentrations of MEs and standard solutions were prepared, and 750 µL of them were mixed with an equal volume (750 µL) of DPPH• solution by vortexing. The reaction mixtures were incubated at room temperature for 50 min. Absorbance values were determined at 517 nm in the UV−Vis spectrophotometer (ATI/Unicam UV2). Triple measurements were conducted throughout the experimental study. The calibration graph was plotted using absorbance versus concentration to determine the unknown sample concentration (IC_50_), and IC_50 _value was identified as the reduced amount of DPPH• by 50%. A low IC_50_ value means high radical scavenging potential and thus high activity.

#### 2.3.3. ABTS•+ radical scavenging assay

Stock ABTS•^+^ solution was prepared by mixing 7 mM ABTS and 2.45 mM potassium persulfate and left in a dark environment for about 16–20 h at room temperature until the day of the analysis. The ABTS•^+^ radical solution was diluted with 60% ethanol to show an absorbance reading of 0.70 ± 0.02 at 734 nm. In this method, 1950 µL ABTS•^+^ radical solution was mixed with 50 µL of the sample, vortexed, and incubated at room temperature for 20 min. Absorbance measurement was carried out at 734 nm (UV−Vis spectrophotometer, ATI/Unicam UV2). The results were given as IC_50 _[33,36].

### 2.4. Determination of total phenolic contents (TPC)

The total phenolics of all MEs were tested with some minor modifications according to the method of Slinkard and Singleton [37,38]. Catechin and gallic acid were used as standard. A 50 μL of the sample was diluted by using 2.5 mL of distilled water. Then, Folin–Ciocalteu reagent (0.2 N) diluted with 250 µL of pure water at intervals of 20 s was added, vortexed, and incubated at room temperature for 3 min. Then, 750 µL (7.5%) Na_2_CO_3_ was added again in 20 s. It was vortexed again by pipetting and left for incubation for 2 h at room temperature. Besides, one blank for each concentration of sample and standard (sample/standard + Folin−Ciocalteu reagent solvent [pure water]) was studied. All the experiments were performed three times. The absorbances were measured at 765 nm (UV−Vis spectrophotometer, ATI/Unicam UV2). A calibration graph was drawn (62.5−1000 μg/mL), and TPC was given as microgram catechin (CE) and gallic acid equivalent (GAE) per mL sample.

### 2.5. Gas chromatography−mass spectrometry (GC–MS) analysis

About 10 mg of root sample was taken and dissolved in hexane. A Thermo Scientific GC−MS was used to identify partial components of EO. The oils were analyzed using the TG−5MS column (film thickness 0.25 µm, 30.0 m × 0.25 mm i.d.). The injection port temperature was set at 250 °C, whereas the oven temperature was arranged as the first temperature was 50 °C with the GC oven temperature was held at 220 °C for 0.67 min and programmed with a rate of 5 °C/min to 250 °C and then held constant at 250 °C for 5 min. The ionization mode was at 70 eV. The carrier gas was helium with a flow rate of 1.0 mL/min. The components were determined by comparison of their relative retention times and mass spectra with those of standards, reported in the literature [39] and available on Wiley and NIST mass spectral libraries. 

### 2.6. Liquid chromatography-tandem mass spectrometry (LC−MS/MS) analysis

Phenolic compounds found in various parts of the *A* . *purpurascens* plant were determined by using LC−MS/MS (Thermo Scientific/TSQ Quantum Access Max) technique. Twenty phenolic compounds were used as standard. Initially, the optimization of the MS program was made, for which different collision energies were used to generate a qualifier ion and a quantifier ion for each standard. Serial dilutions of the standards (0.25−0.5−1−2−4−6 mg/L) were used to obtain a linear standard curve (r^2 ^> 0.99). *A* . *purpurascens* phenolics were identified by matching the retention time and MS spectra with those of the standards.

A reversed-phase Hypersil™ ODS C_18_ column (4.6 × 250 mm 5µm) was used, and 0.1% formic acid in water (A) and 100% methanol (B) were used as the mobile phase solutions with a flow rate of 0.7 mL/min. A 20-μL injection volume and 30 °C column temperature were used. The gradient program included an initial 0–1 min of 100% A and the following compositional changes: 1−22 min, 100% A; 22−25 min, 5% A; 25−30 min 100% B. Mass spectrometry signals were acquired by maintaining the temperature for capillary at 300 °C and for vaporizer at 350 °C; spray voltage of positive and negative polarity was set to 4000 and 2500 V; the pressure of sheath gas and aux gas were kept at 30 arb and 13 arb, respectively; discharge current 4 μA. 

### 2.7. Statistical analysis

Antioxidant test results and total phenolic contents were statistically analyzed by using a one-way analysis of variance ANOVA with Tukey post hoc test using SPSS 22.0 software. Test results were expressed as mean ± standard error (SD) of three experiments, and the differences were considered significant at p < 0.01.

## 3. Results and discussion

### 3.1. Antioxidant activity and total phenolic content of A. purpurascens

There are many antioxidant assays in the literature based on methodological differences to screen antioxidant capacities of samples from natural sources, including extracts and essential oils from plants. In this study, DPPH•, ABTS•^+^,and FRAP methods were used to determine the antioxidant potentials of *A* . *purpurascens* MEs and EO (Table 1). 

**Table 1:** Total phenolic content and antioxidant activity of root, stem, seed, and leaf extracts and root essential oil of A. purpurascensx and standards.

Sample	TPCy(GAE, μg/mL)	Antioxidant activity		
		DPPH• scavenging(IC50, mg/mL)	ABTS•+ radical scavenging(IC50, mg/mL)	FRAPy(TEAC, μM)
Root	438.75 ± 16.39d	0.06 ± 0.002a	0.05 ± 0.0001a	821.04 ± 15.96d
Stem	68.33 ± 1.90a	1.23 ± 0.001d	0.19 ± 0.003d	86.88 ± 5a
Seed	128.33 ± 5.05b	0.35 ± 0.003c	0.18 ± 0.004c	132.5 ± 4.50b
Leaf	408.75 ± 8.75c	0.09 ± 0.0007b	0.09 ± 0.003b	487.50 ± 13.62c
Essential oil	NT	2.95 ± 0.084	NT	143.33 ± 5.63
Trolox	NT	0.002 ± 5.7735E-05	0.003 ± 0.0001	#
BHT	NT	0.008 ± 1.7321E-05	0.0005 ± 1.7321E-05	NT
Quercetin	#	NT	0.0014 ± 0.00001	NT
Gallic Acid	#	NT	0.0006 ± 1.5275E-05	NT

The ABTS•^+^ and DPPH• assays result expressed as IC_50_ means the effective concentration of test samples required for 50% antioxidant activity under the experimental conditions. Lower IC_50_ values indicate higher radical scavenging activity. The root extract showed higher values than other parts. The root methanolic extract of *A. purpurascens* demonstrated the highest ABTS•^+^ and DPPH• radical scavenging activities (IC_50_: 0.05 ± 0.0001^a^ mg/mL and IC_50_: 0.06 ± 0.002^a^ mg/mL, respectively) while the stem methanolic extract showed the lowest antioxidant activities (IC_50_: 0.19 ± 0.003^d^ mg/mL and IC_50_: 1.23 ± 0.001^d^ mg/mL, respectively). Moderate antioxidant activity was observed in seed and leaf extract (IC_50_: 0.35 ± 0.003^c^ and 0.09 ± 0.0007^b^ mg/mL for DPPH• and IC_50_: 0.18 ± 0.004^c^ and 0.09 ± 0.003^b^ mg/mL for ABTS•^+^, respectively). Moreover, the root EO of *A. purpurascens* displayed DPPH• scavenging activity (IC_50_: 2.95 ± 0.084 mg/mL). Essential oil and all MEs showed significant radical scavenging activities, though lower than those of standard antioxidants (Table 1). 

The FRAP test results were expressed in comparison to the activity of Trolox, and the Trolox equivalent antioxidant capacity (TEAC, μM) values obtained from the calibration graph were used to express antioxidant potentials. The higher TEAC values in the FRAP test indicate better antioxidant activity. The FRAP activity values of the EO and MEs of different parts *A. purpurascens* were in the range of 86.88 ± 5^a^−821.04 ± 15.96^d^ µM TEAC. While the highest FRAP value of all samples was found in root extract (821.04 ± 15.96^d^ µM TEAC), the lowest value was in stem extract (86.88 ± 5^a^ µM TEAC). According to all antioxidant assays, all extracts displayed an antioxidant activity with the order of activity root > leaf > seed > stem. 

Significant differences were observed in the total phenolic content and antioxidant activities of the root, stem, seed, and leaf parts of *A. purpurascens* (Table 1). The root extract was observed to have the highest total phenolic content (438.75 ± 16.39^d^ GAE, µg/mL) compared with the stem, seed, and leaf parts. The stem extract had a significantly lower phenolic content (68.33 ± 1.90^a^ GAE, µg/mL) than the extracts from other plant parts. The order of the total phenolic content of *A. purpurascens* MEs was root > leaf > seed > stem. Good positive correlations were observed between the results of the phenolic content and antioxidant assays (ABTS•^+^, DPPH•, and FRAP; r^2^ values were 0.8927, 0.9212, and 0.8587, respectively). 

Although the antioxidant potentials of some *Angelica* species essential oil or extracts have been reported in the literature, it was found only one study reporting on the antioxidant activity of *A. purpurascens* . Karakaya et al. (2020) evaluated MEs of different parts of (root, fruit, and aerial) *A. purpurascens* in terms of antioxidant activity [16]. There was no study in the literature attempting to determine the antioxidant capacity of *A. purpurascens* EO using the DPPH•, ABTS•^+^,and FRAP methods. In this study, the antioxidant activity of *A. purpurascens* EO was also determined for the first time. In a previous research, DPPH• scavenging activity for the ME of *A. gigas* aerial part was a dose-dependent antioxidant activity, and it was lower than that of synthetic antioxidants vitamin C and BHT [40]. Similarly, *A. glauca* oil was noted to increase the DPPH• scavenging capacity in a concentration-dependent manner with an IC_50 _value of 32.32 µg/mL, but showed lower activity compared to BHT [41]. Moreover, Pervin et al. (2014) reported that *A. dahurica* root extracts showed a dose-dependent increasing DPPH• and ABTS•^+ ^scavenging activities [42]. Roh and Shin (2014) demonstrated that *A. koreana* root EO and its two main components showed less scavenging activity than butylated hydroxyl anisole (BHA) at 1 mg/mL [43]. In some previous studies, *A. archangelica* L. seed EO [44] and *A. sinensis* extracts [45] exhibited moderate DPPH• scavenging activity. Moreover, ME of *A. officinalis* L. fruits did not have radical scavenging activity against DPPH• while it had moderate FRAP activity [46]. Leaf extract of *A. keiskei* showed significant DPPH• scavenging activity close to the rutin standard [47]. The MEs of two species of *Angelica* ( *A. pancicii* and *A. sylvestris* ) exhibited a positive correlation between antioxidant activity and polyphenol content [48]. *A. sylvestris* var. *sylvestris* EOs from dried roots, leaves, flowers, and fruits mentioned by Ağalar et al. (2020) had similarly low antioxidant activity [23]. Finally, Zhang et al. (2020) reported that among the antioxidant activities of different solvent extracts of *A. amurensis* root, methanol, and ethanol extracts exhibited high antioxidant activity [49]. In this study, *A. purpurascens* MEs and EO had low antioxidant activity, similar to the activity results of other *Angelica* species mentioned in the literature. Although the antioxidant activity of *A. purpurascens* MEs and EO was weaker than that of the standard compounds, its use would prevent the toxicity problems of the synthetic standards. However, further studies are recommended before the usage of *A. purpurascens* MEs and EO as antioxidant additives.

### 3.2. Identification and quantification of phenolic compounds in A. purpurascens

The identification of the phenolics was accomplished by comparing retention times and MS fragments with those of reference standards. Molecular ions of phenolic standard compounds were determined with both negative and positive ion modes in LC−MS/MS (Table 2). In LC−MS/MS analysis, 20 phenolic compounds were identified and quantified (Table 3). The seed extract of *A. purpurascens* was found to have the highest value in terms of phenolic compound concentration compared with the extracts from other plant parts. The major phenolic compound of seed extract was found as ferulic acid (244.39 μg/g extract). Benzoic acid (138.18 μg/g extract), oleuropein (78.04 μg/g extract), and rutin (31.21 μg/g extract) were found as the most abundant phenolic compounds in the stem, root, and leaf extracts, respectively. Ferulic acid, benzoic acid, and rutin were found in one part of the plant. In addition, pyrogallol, gallic acid, chlorogenic acid, myricetin, and ellagic acid were found in different amounts in one part of the plant. 4-Hydroxybenzaldehyde, vanillin, syringic acid, and hesperidin phenolic compounds were detected in all plant parts. 

**Table 2:** Name of phenolic standards, precursor and fragment(s) ions, polarity, and the optimized ion mode for LC−MS/MS.

No	Compound	Precursorion [m/z]	Fragment(s)ions [m/z]	Energy	Polarity
1	Pyrogallol	124.86	79.369.3	2320	Neg.
2	Gallic acid	169.7	126.280.5	1625	Neg.
3	Protocatechuic aldehyde	136.9	108.292.24	2525	Neg.
4	Chlorogenic acid	353.4	192.186.5	2143	Neg.
5	Caffeic acid	179.7	136.2135.2	1827	Neg.
6	4-Hydroxybenzaldehyde	121.9	121.193.5	2025	Neg.
7	Vanillin	150.91	136.192.3	1623	Neg.
8	Syringic acid	183.07	123.277.3	1323	Neg.
9	Syringaldehyde	180.88	166.1151.1	1623	Neg.
10	Ferulic acid	194.24	179.94135.35	1518	Neg.
11	Hesperidin	609.13	301.1164.1	2659	Neg.
12	Luteolin-7-Glucoside	446.89	284285	4540	Neg.
13	Rutin	609.37	300.6301.7	3834	Neg.
14	Oleuropein	539.1	275.8377.5	2216	Neg.
15	Benzoic acid	120.98	77.3	13	Neg.
16	Resveratrol	228.98	135.1107.2	1422	Pos.
17	Myricetin	316.91	179.2137.1	2225	Neg.
18	Apigenin	268.86	149.1117.2	2740	Neg.
19	Naringenin	273	153147.1	2420	Pos.
20	Ellagic acid	300.91	145.118.1	3928	Neg.

**Table 3:** Phenolic components of different parts of A. purpurascens.

Compound	Concentration (μg/g extract)			
	Root	Stem	Seed	Leaf
Pyrogallol	1.99 ± 0.13	nd	nd	nd
Gallic acid	nd	nd	9.63 ± 0.62	nd
Protocatechuic aldehyde	12.59 ± 0.81	nd	8.18 ± 0.52	nd
Chlorogenic acid	nd	nd	8.45 ± 0.47	nd
Caffeic acid	5.26 ± 0.34	nd	2.59 ± 0.17	5.08 ± 0.32
4-Hydroxybenzaldehyde	7.89 ± 0.51	42.84 ± 2.74	6.93 ± 0.44	7.39 ± 0.47
Vanillin	11.85 ± 0.76	68.69 ± 4.40	10.90 ± 0.70	2.98 ± 0.19
Syringic acid	5.79 ± 0.37	16.89 ± 1.08	1.07 ± 0.07	3.59 ± 0.23
Syringaldehyde	nd	14.16 ± 0.91	nd	4.20 ± 0.27
Ferulic acid	nd	nd	244.39 ± 15.64	nd
Hesperidin	15.32 ± 0.98	5.11 ± 0.33	7.59 ± 0.49	18.86 ± 1.21
Luteolin-7-Glucoside	nd	nd	0.87 ± 0.06	2.85 ± 0.18
Rutin	nd	nd	nd	31.21 ± 2.00
Oleuropein	78.04 ± 4.99	nd	13.73 ± 0.88	nd
Benzoic acid	nd	138.18 ± 8.84	nd	nd
Resveratrol	nd	1.90 ± 0.12	1.63 ± 0.10	nd
Myricetin	nd	nd	0.95 ± 0.06	nd
Apigenin	1.20 ± 0.08	5.81 ± 0.37	nd	nd
Naringenin	0.62 ± 0.04	1.30 ± 0.08	1.50 ± 0.10	nd
Ellagic acid	22.46 ± 1.44	nd	nd	nd

Oleuropein, which is generally found in some Oleaceae species, has been documented by in vitro and in vivo studies to have antitumor, antifungal, antimicrobial, anticancer, and cardioprotective properties, besides its strong antioxidant activity as a free radical scavenger [50]. In this study, oleuropein, which is abundant in *A. purpurascens* root, may be responsible for the high antioxidant activity. In a previous paper involving the phenolic composition of *A. purpurascens* , four coumarin derivatives (ostruthol, phellopterin, xanthotoxin, and biakangelicin) were isolated from roots, and the last three of them were isolated for the first time [25]. The previous study of characterization of phenolic compounds has led to the identification of furocoumarins, including imperatorin, phelloptorin, and isoimperatorin, in the roots of *A. dahurica* by HPLC/DAD/ESI-MS [51]. Coumarins including psoralen and xanthontoxin, and chalcones have been reported as phenolic compounds found in *A. keiskei* [52]. 

Analysis of phenolic compounds in some *Angelica* species has been previously reported, but quantitative analysis of methanol extracts from different parts of *A. purpurascens* has not been reported previously. This novel study is the first report of the identification and quantification of phenolic components found in all four parts of *A. purpurascens* , root, stem, leaf, and seed, by LC−MS/MS.

### 3.3. GC−MS analyses of essential oil of A. purpurascens root

Gas chromatography-mass spectrometry (GC−MS) is one of the most widely used methods in determining the chemical composition of EOs [53,54]. According to the literature, the EOs of different *Angelica* species grown in different geographical regions have been obtained by various extraction techniques such as hydrodistillation, steam distillation, supercritical liquid extraction, and solvent-free solid injection. Besides, it was reported that the EOs of *Angelica* species show various biological activities, including antibacterial, antifungal, insecticidal activities, and pronounced antioxidant activity due to their volatile compositions [29].

The EO composition of *A. purpurascens* root is listed in Table 4, and twenty-nine components of *A. purpurascens* root essential oil were identified, representing 97.10% of the total volatiles. The components were divided into five classes: monoterpene hydrocarbons (30.98%), monoterpeneoids (1.23%), sesquiterpene hydrocarbons (25.78%), sesquiterpenoids (38.99%), and terpene-like compounds (0.12%). The main components were sesquiterpenoid-like compounds (38.99%) [(α-bisabolol (22.93%) and cubebol (14.39%) ]. Monoterpene hydrocarbons were present at a rate of 30.98% and α-pinene (11.63%), α-limonene (9.41%), sabinene (4.48%) were the major components among the monoterpene hydrocarbon components. Sesquiterpene hydrocarbons were present at 25.78%, the most important of which are aromadendrene-dehydro (4.64%), β-elemene (4.56%), and germacrene-D (4.47%), respectively.

**Table 4:** The EO composition of A. purpurascens root.

Compoundsa	Area (%)	Exp. RIb	Lit.RI
α-Pinene	11.63	930	939
Camphene	1.10	954	954
Sabinene	4.48	973	975
β-Myrcene	0.96	990	991
α-Phellandrene	0.42	1000	1003
α-Terpinene	0.65	1015	1017
α-Limonene	9.41	1029	1029
γ-Terpinene	1.97	1060	1060
Terpinolene	0.36	1085	1089
6-Camphenone	0.13	1095	1097
6-Camphenol	0.09	1112	1114
Terpinen-4-ol	1.01	1175	1177
Bornyl acetate	0.12	1285	1289
δ-Elemene	0.18	1334	1338
α-Ylangene	0.08	1376	1375
β-Elemene	4.56	1390	1391
Z-Caryophyllene	0.51	1405	1409
γ-Elemene	2.00	1438	1437
Aromadendrene-dehydro	4.64	1462	1463
9-epi-(E)-Caryophyllene	1.10	1465	1466
Germacrene-D	4.47	1485	1485
Viridiflorene	1.81	1490	1497
α-Muurolene	1.70	1498	1500
β-Bisabolene	2.81	1505	1506
Cubebol	14.39	1510	1515
Elemol	1.18	1545	1550
Germacrene B	1.92	1560	1561
α-Bisabolol	22.93	1685	1686
Iso-Longifolol	0.49	1728	1730
Constituents	Contentb(%)		
Monoterpene hydrocarbons	30.98		
Oxygenated monoterpenes	1.23		
Sesquiterpene hydrocarbons	25.78		
Oxygenated sesquiterpenes	38.99		
Terpene related compound	0.12		
Total	97.10		

In a previous study, the chemical composition of *A. purpurascens* fruits was reported to be rich in isooxypucledanin, coumarin (agacillin), and β-sitosterol [30,55]. Acyl- and pyranocoumarins were observed in the ethanol extracts of root and rhizomes [56]. The composition of the EOs obtained from the fruit of *A. purpurascens* by hydrodistillation was analyzed by GC and GC−MS [57]. Başer et al. identified 119 compounds, representing 85.2% of the EO, and bicyclogermacrene (12.0%), β-phellandrene (7.1%), spathulenol (6.9%), and kessan (6.6%) were defined as the main components. Another study based on the GC and GC−MS analyses reported that the oil of *A. purpurascens* fruit exhibited different compositional profiles because of different extraction methods [hydrodistillation (HD), microdistillation (MD), and microsteam distilled solid-phase microextraction (MSD−SPME)] [31]. In the study, the authors noted that the major components of HD− and MD−oils were monoterpenes α-phellandrene (32% and 27%), β-phellandrene (22.8% and 19.8%), limonene (5.3% and 4.5%), *p* -cymene (3.7% and 2.8%), and α-pinene (3.2% and 2%, respectively). The hydrodistillation method is the most effective in the isolation of monoterpenes. The highest amount of monoterpenes and their oxygenated forms were obtained with HD, MD, and MSD-SPME techniques as 72%, 62%, and 44.5%, respectively. In another study, *A* . *purpurascens* was harvested at the flowering stage, and its EO was obtained from its aboveground parts, and more than 26 compounds were detected. β-phellandrene (20.1%) and β-caryophyllene (11.3%) were found as the main components [58]. As a result, the major and other components in the essential oils obtained from the plant differ due to reasons such as the harvesting time of the plant, geographical and climatic conditions, and different parts of the plant [23,57].

## 4. Conclusion

The root essential oil and the methanol extracts of *A. purpurascens* exhibited a remarkable antioxidant potential. The strong antioxidant activity of *A. purpurascens* with high total phenolic content indicated a great potential for its use in the production of functional foods. The biochemical compositional data thus obtained for the extracts from different parts of *A. purpurascens* (root, stem, seed, and leaf) and essential oil can form a background for further investigations to develop new formulations or products by the use of *Angelica* species. 
